# A Natural Combination Extract of *Viscum album* L. Containing Both Triterpene Acids and Lectins Is Highly Effective against AML *In Vivo*

**DOI:** 10.1371/journal.pone.0133892

**Published:** 2015-08-05

**Authors:** Catharina I. Delebinski, Monika Twardziok, Susann Kleinsimon, Florian Hoff, Katharina Mulsow, Jana Rolff, Sebastian Jäger, Angelika Eggert, Georg Seifert

**Affiliations:** 1 Department of Paediatric Oncology/Haematology, Otto Heubner Centre for Paediatric and Adolescent Medicine (OHC), Charité –Universitaetsmedizin, Berlin, Germany; 2 Department of Cell Biology and Cell Pathology, Philipps University, Marburg, Germany; 3 Institute of Pharmacy, Freie Universität, Berlin, Germany; 4 EPO GmbH, Berlin, Germany; 5 Birken AG, Niefern-Oeschelbronn, Germany; University of Windsor, CANADA

## Abstract

Aqueous *Viscum album* L. extracts are widely used in complementary cancer medicine. Hydrophobic triterpene acids also possess anti-cancer properties, but due to their low solubility they do not occur in significant amounts in aqueous extracts. Using cyclodextrins we solubilised mistletoe triterpenes (mainly oleanolic acid) and investigated the effect of a mistletoe whole plant extract on human acute myeloid leukaemia cells *in vitro*, *ex vivo* and *in vivo*. Single *Viscum album* L. extracts containing only solubilised triterpene acids (TT) or lectins (viscum) inhibited cell proliferation and induced apoptosis in a dose-dependent manner *in vitro and ex vivo*. The combination of viscum and TT extracts (viscumTT) enhanced the induction of apoptosis synergistically. The experiments demonstrated that all three extracts are able to induce apoptosis via caspase-8 and -9 dependent pathways with down-regulation of members of the inhibitor of apoptosis and Bcl-2 families of proteins. Finally, the acute myeloid leukaemia mouse model experiment confirmed the therapeutic effectiveness of viscumTT-treatment resulting in significant tumour weight reduction, comparable to the effect in cytarabine-treated mice. These results suggest that the combination viscumTT may have a potential therapeutic value for the treatment AML.

## Introduction

Acute myeloid leukaemia (AML) is the second most common type of leukaemia in the US and Germany. The incidence rises with age and less than 10% of patients are children. Prognosis, remission and mortality rate also depend strongly on age. Approximately two-thirds of adult patients achieve complete remission, but most of them relapse within 3 years and die within 5 years [1]. Increasing understanding of AML´s biology has resulted in novel therapeutic agents, several of which have been evaluated in clinical trials. In addition to classic anti-tumour agents, compounds of natural products such as triterpene acids or flavonoids show promising effects [2–5].

The European white berry mistletoe, *Viscum album* L. *(Loranthaceace)*, is often used in complementary cancer therapy [6, 7]. *Viscum album* L. contains a variety of many biologically active hydrophilic and lipophilic substances as viscotoxins, lipids, polysaccharides, flavonoids, alkaloids, triterpene acids and various other (glyco-) proteins [8–11]. Among the glycoproteins, mistletoe lectins I-III (ML) are the most important and best studied mistletoe substances today and have been identified as the predominant cytotoxic components in aqueous *Viscum album* L. extracts [12].

Commercial standardised *Viscum album* L. extracts (VAEs) are water-based and contain hydrophilic cytotoxic and immune-modulatory proteins such as mistletoe lectins and viscotoxins [13–16]. They are known to stimulate the immune system by activating leukocytes resulting in cytokine release, inhibition of cell proliferation and induction of apoptosis *in vitro* and *in vivo* [17, 18]. ML-induced apoptosis is primarily triggered by PI3K/Akt-, MAPK-, TLR-signalling resulting in the activation of caspases [19–22]. Its cytotoxic and anti-metastatic effect has been demonstrated in different solid tumours and leukaemia cell lines *in vitro* and *in vivo* [23–26].

Mistletoe constituents of the family of pentacyclic triterpene acids (oleanolic acid, betulinic acid, ursolic acid) also possess cytotoxic anti-cancer activity but due to their low solubility they do not occur in aqueous mistletoe extracts [27–30]. Preclinical studies have proven the anti-inflammatory and anti-carcinogenic properties of triterpene acids such as betulinic acid (BA) or oleanolic acid (OA) [31–33]. Moreover, OA and its derivatives have been shown to induce apoptosis in various malignant cells [32, 34–37]. Similar to ML-induced apoptosis the main described pathways of OA-induced apoptosis include the Akt-, MAPK-, ERK-, JNK-signalling pathways [38–41]. Inhibition of cell growth and induction of apoptotic cell death has also been shown in leukaemia cells [42, 43]. The anti-tumour effects of BA and ursolic acid are comparable to those of OA [44, 45]. New results indicate a synergistic effect of combined oleanolic and ursolic acids in human melanoma cell lines *in vitro* and *in vivo* [46].

It is a classical assumption of phytopharmacology that a naturally occurring combination is sometimes more beneficial than single compounds. A good example of such a combinatory effect is the pharmacological property of St. John's wort (*Hypericum perforatum*). Mistletoe lectins and triterpene acids both occur naturally in the mistletoe plant. We have already demonstrated the therapeutic effect of a combination *Viscum album* L. extract containing mistletoe lectin I and solubilised triterpene acids (viscumTT) in pre B-acute lymphoblastic leukaemia (B-ALL) *in vitro* and *in vivo* [47]. Moreover, viscumTT demonstrated an amplified anti-tumour effect on murine melanoma *in vivo* [48].

For the combination viscumTT the mistletoe triterpene acids (mainly oleanolic and betulinic acid) were solubilised by using cyclodextrins, resulting in a plant extract with high levels of OA and MLs in combination [47, 49, 50].

The aim of the present study was to examine the therapeutic potential of viscumTT as cancer therapy in AML. In addition to the effects of defined single extracts containing either ML-I (viscum) or triterpenes (TT) the cytotoxic effects of viscumTT were characterized in two leukaemia cell lines and two patient samples. Induction of apoptosis was determined by flow cytometry using Annexin V/Propidium Iodide (PI), JC-1 and active caspase staining. Apoptosis associated proteins were analyzed by Western blot analysis. Finally, *in vivo* anti-cancer efficacy was examined using a human AML mouse model.

## Materials and Methods

### Ethic statement

Animal experiments were performed according to German legislation on the care and use of laboratory animals (Tierschutzgesetz) and with a formal approval of the ethical approval board of the "Landesamt für Gesundheit und Soziales Berlin (LAGeSo)—the responsible authority.

### Material and reagents

RPMI 1640, penicillin, streptomycin and PBS were purchased from Gibco, Lifetechnologies (Darmstadt, Germany). FCS was purchased from Biochrom (Berlin, Germany). RIPA buffer, protein inhibitors, molecular mass standards for SDS-PAGE, DMSO, TX-100, Histopaque, Sodium dodecyl sulphate (SDS), 5,6,6-tetrachloro-1,1,3,3-tetraethylbenzimidazol-carbocyanine iodide (JC-1), carbonyl cyanide 3-chlorophenylhydrazone (CCCP) and propidium iodide (PI) were obtained from Sigma-Aldrich (Munich, Germany). Tween, Sulphuric acid, acrylamide and dithiotreitol were purchased from Carl Roth GmbH, (Karlsruhe, Germany). Ammonium persulfate and N,N,N,N-tetramethylenediamine were obtained from BioRad (Munich, Germany). 3,3’,5,5’-tetramethylbenzidine was purchased from eBioscience Inc. (San Diego, USA). Following primary antibodies were used: caspase-3, poly (ADP-ribose) polymerase (PARP), claspin, survivin, bcl-2, cytochrome c (Cell Signaling Technology, Danvers, USA); p53 (Santa Cruz biotechnology, Santa Cruz, CA, USA); X-chromosome-linked IAP (XIAP) and Annexin V-APC (BD Bioscience, Heidelberg, Germany); ß-actin-peroxidase antibody (Sigma-Aldrich, Munich, Germany). The Cytotoxicity Detection Kit was purchased from Roche (Grenzach-Wyhlen, Germany).

### *Viscum album* L. extracts

*Viscum album* L. extracts were kindly supplied by Birken AG (Niefern-Oeschelbronn, Germany). Preparation of *Viscum album* L. extracts were performed as described before [47, 51].

Sprouts from *Viscum album* L. were harvested from apple trees (*Malus domestica* Borkh.) and identified by the co-author S. Jaeger. Two different extracts were prepared to obtain the oleanolic acid-containing TT- extract and the mistletoe lectin containing viscum- extract. The combination of both extracts is further described as viscumTT.

Preparation of the oleanolic acid containing TT- extract: Oleanolic acid (OA) was extracted from dried plant material resulting in a dry extract containing 69.4% OA and 6.9% betulinic acid [52]. 100 mg of these triterpene acids were mixed with 2-HP-β-cyclodextrins (2-hydroxypropyl-β-cyclodextrin) and suspended in water. The dried (105°C) mixture was pestled, the resulting powder suspended in sodium dihydrogen phosphate buffer (30 mM, pH 8.0) and the mixture sonicated for 30 min. After adjusting the pH to 7.5 (100 mM phosphoric acid) the volume was made up to 25 mL (30 mM sodium phosphate buffer pH 7.5). After filtration OA was quantified in triplicate in each solution using GC-FID and external calibration with OA as reference substance (>97%, Extrasynthese, Genay Cedex, France) [30].

Preparation of the mistletoe lectin-containing viscum- extract: For the aqueous extract (lectin and viscotoxin-containing viscum) plant material was milled under liquid nitrogen using a cryo mill (Retsch, Germany) and extracted using ascorbate phosphate buffer (30 mM sodium phosphate, 3.4 g/L ascorbic acid, pH 9.1) resulting in a filtered (0.22 μm) extract pH 7.5. Preparation of viscumTT: *Viscum album* L. extracts (VAE) are combined to viscumTT by mixing of both extracts. The concentrations of the different extracts are described in Table 1.

**Table 1:** *Viscum album* L. extracts.

	2-HP-CD [mg/mL]	OA [mg/mL]	BA [mg/mL]	ML [μg/mL]
**TT**	**230**	**4.0**	**0.38**	**-**
**viscum**	**-**	**-**	**-**	**3.7**
**viscumTT**	**230**	**+**	**+**	**+**

Table 1 gives a schematic presentation of the concentrations of the applied *Viscum album* L. extracts TT, viscum and viscumTT. OA = oleanolic acid; ML = mistletoe lectin-I, BA = betulinic acid; CD = 2-hydroxypropyl-β-cyclodextrin.

### Measurement of mistletoe lectin I concentration in *Viscum album* L. extracts

Intact mistletoe lectin T (A+B chain) in the viscum extract was analyzed by ELISA [53]. The microtiter wells (Nunc Immuno Plate Maxisorb) were coated with 10μg/100 μl asialofetuin type I (0.1 mg of ASF-I/1 ml PBS, Sigma-Aldrich, St. Louis, USA) incubating for 30 min, then washed three times with PBST. Afterwards, the plate was blocked with BSA 3% /PBST for 1 h, followed by three wash steps with PBST. 100 μl samples and five standard mistletoe solutions were incubated for 1 h. After three wash steps, 100 μl POD labelled (1:4000) detection antibody AB-5H8 (Sifin diagnostic GmbH, Berlin, Germany) was incubated for 1 h, followed by three wash steps with PBST. 50 μl/well of substrate solution (3,3’,5,5’-tetramethylbenzidine) was incubated in the dark for 20 min. The reaction was stopped by 50 μl/well of 1 M sulphuric acid. Absorption was measured at 450 nm (reference wavelength: 620 nm). All incubation steps were performed at room temperature shaking at 200 rpm. Mistletoe lectin I stock solution 5.3 mg/ml in 3 M ammonium sulphate was provided by Dr. U. Pfueller, Universitaetsklinikum Hamburg-Eppendorf, Institute of anatomy II, Experimental morphology, Germany.

### Cell culture

The human acute myeloid leukemia cell lines U937 and HL-60 were obtained from the German Collection of Microorganism and Cell Cultures (DSMZ, Braunschweig, Germany). The cell lines were maintained as the suppliers recommended in RPMI 1640 with supplements. For the assays U937 cells were seeded at a density of 2x10^6^ cells and HL-60 to 4x10^5^ cells per 6-well plate immediately before addition of defined VAEs. Unless otherwise stated, cells were incubated for 18 h with depicted concentrations of VAE.

### Cell proliferation and measurement of LDH

Cell proliferation was determined by using CASY Cell Counter and Analyser System of Schaerfe System GmbH (Reutlingen, Germany). Settings were specifically defined for the requirements of the used cells.

The early cytotoxicity of different VAE concentrations was measured after 2 h of incubation using the Cytotoxicity Detection Kit that quantifies lactate dehydrogenase (LDH) according to the manufacturer’s protocol.

### PI and Annexin V binding assay

Cells were incubated for 18 h with depicted VAE. After incubation, cells were stained as described before [47]. The results were evaluated with FlowJo Software (TreeStar, Ashland, USA).

### Analysis of mitochondrial membrane potential (ΔΨ_m_)

The mitochondrial membrane potential was assessed using 5,5,6,6-tetrachloro-1,1,3,3-tetraethylbenzimidazol-carbocyanine iodide (JC-1) and flow cytometric analysis after VAE treatment as described before [47].

### Protein extraction and Western blot analysis

After VAE incubation, HL-60 cells were washed twice with PBS and lysed in RIPA buffer containing protease inhibitors. Protein lysates were cleared of cellular debris by centrifugation for 10 min at 13 000 rpm at 4°C. The protein concentration was determined using Bradford Reagent (Bio-Rad, Munich, Germany). Equivalent amounts of proteins were separated by SDS-PAGE and transferred to nitrocellulose membranes (Bio-Rad, Munich, Germany). Primary antibody incubations were performed overnight at 4°C. Detection was performed with HRP-conjugated secondary antibodies (Bio-Rad, Munich, Germany) which were visualized by ECL (Thermo Fisher Scientific, Bonn, Germany) and Molecular Imager ChemiDoc (Bio-Rad, Munich, Germany).

### Preparation of mitochondrial proteins

After VAE treatment, the cytosolic fractions were isolated as described before [47]. SDS-PAGE was performed with the cytosolic fraction, transferred to nitrocellulose membrane and incubated with monoclonal cytochrome c antibody. Quantification was performed as described before [47, 54]. Relative density of Ctrl = 1.

### Proteome Profiler Human Apoptosis Array

HL-60 cells were treated with depicted VAE concentrations for 18 h. Cell lysates were incubated with a human apoptosis array membrane, detecting the relative expression of 35 apoptosis-related proteins per sample according to the manufacturer’s protocol (R&D, Minneapolis, MN, USA). Immunoreactive dots were visualized using Molecular Imager ChemiDoc (Bio-Rad, Germany). Each experiment was carried out in duplicate (n = 2).

### Measurement of caspase activity

Activity of caspases-8 and -9 was measured by fluorescent caspase staining kit according to the manufacturer’s protocol (Promokine, Heidelberg, Germany).

For caspase inhibitor assay, cells were pre-incubated with 100 μM z-VAD-fmk, z-IETD-fmk or z-LEDH-fmk for 1 h [21, 55]. After additional incubation with VAE for 18 h, induction of apoptosis was determined with Annexin V-APC / PI via FACS analyses. DMSO was added to extracts as solvent control.

### Measurement of TRAIL-receptor

Activity of TRAIL-receptor-1 and -2 (ALEXIS, Biochemicals Corporation, San Diego, USA) was measured by FITC-labeled monoclonal antibody and flow cytometry. HL-60 cells were treated with distinct VAE concentrations for 18 h, washed twice with PBS + 5% FCS and incubated with 5 μg/mL specific antibody for 30 min on ice. After incubation, cells were washed twice with PBS and analyzed by flow cytometry.

### Animals

Female NOD/SCID/IL2rg mice were obtained at eight weeks of age from Charles River Laboratories (Sulzfeld, Germany). They were housed in the institution in a specific pathogen-free (SPF) facility, maintained under pathogen-free conditions, fed autoclaved standard diet purchased from Sniff (Soest, Germany) and given acidified drinking water *ad libitum*. All animal experiments were performed in accordance with the UKCCCR regulations for the Welfare of animals. Surgery was performed under anesthesia, and all efforts were made to minimize suffering. The health of the mice was examined at the start of the experiment and daily during the experiment. Thereby, clinical observations used in cancer research studies as described by Montgomery *et al*. were applied [56]. Body weight was measured twice a week. As soon as body weight loss or clinical criteria were observed, animals were sacrificed. The experiment was terminated on day 27 for all animals. Mice were sacrificed by cervical dislocation.

### Transplantation of AML and experimental procedures

The systemic leukaemia was induced by injection of 1x10^6^ human HL-60 cells in NOD/SCID/IL2rg mice intravenously (i.v). The mice were divided into six groups of ten mice and were medicated with dose-escalation regimen of 20/40/60 mg/kg oleanolic acid (TT), 0.3/0.4/0.5 μg/kg mistletoe lectin I (viscum) or the combination viscumTT. Each concentration were given twice every third day (i.v.), starting on day three after tumor cell injection. One group received the cytostatic cytarabine 100 mg every third day, four doses. One group was treated with viscumTT plus cytarabine. The control group received an equal amount of cyclodextrins. Body weight was measured twice per week and the mice were carefully monitored for symptoms of toxicity. Mice were sacrified on day 27 and toxicity was measured by autopsy and HE staining of the liver, spleen and bone marrow. Student´s t-test was applied to determine the differences between the groups.

### AML samples

Bone marrow aspirats were obtained from two patients with acute myeloid leukaemia, patient 1 (m; 17 years, FAB M0); patient 2 (m, 10 years, FAB M4, deletion 7q und monosomie 7, r(21)). The use of the patient sample was in acceptance with a positive vote of the ethics committee of the Charité and the ethical standards of the Helsinki Declaration as revised in 2000. Diagnosis was confirmed by immunophenotyping of leukaemia cells according to Bene *et al*. [57]. Mononuclear cells were separated by centrifugation with histopaque. Cells were diluted to a density of 5x10^5^/ml in RPMI 1640 + 10% FCS, 1% penicillin and streptomycin. VAEs were added for 18 h.

### Statistical analyses

The fractional product (Fp) of Webb was used to determine the synergistic effect of viscumTT on induction of apoptosis [58]. The formula was used as shown before [47].: Student´s t-test was applied to determine the differences between VAE-treated animals and control group. The significance level was set to p ≤ 0.05.

### Data set underlying the results

**Fig 1:**
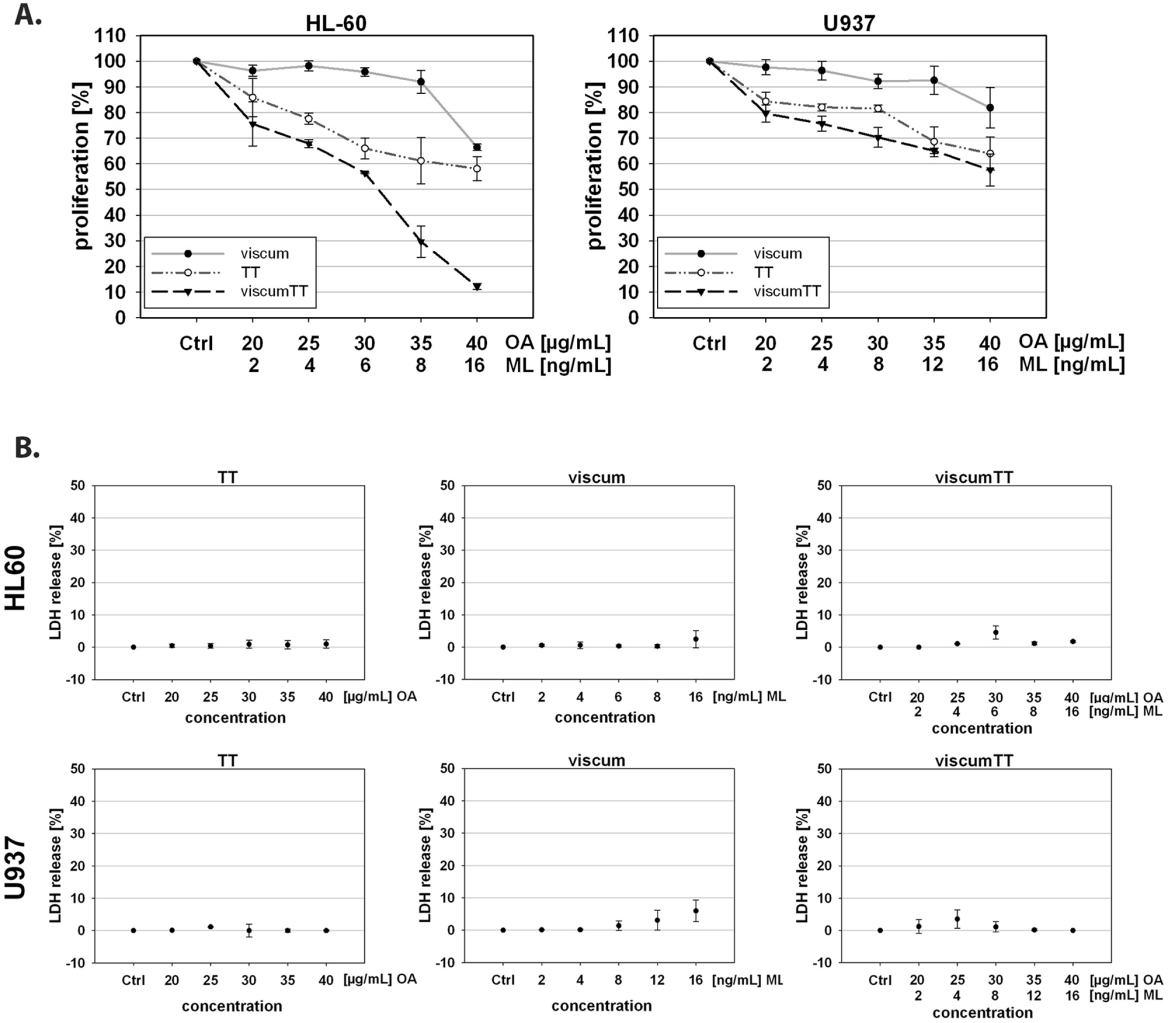
Effects of VAE on viability and proliferation rate of U937 and HL-60 cells. **A.** U937 and HL-60 cells were incubated with increasing doses of VAE (TT, viscum or viscumTT) for 18 h. Proliferation was measured by the CASY Cell Counter System. VAE treated cells show a dose-dependent inhibition of proliferation (n = 3). **B.** U937 and HL-60 cells were incubated with increasing concentrations of VAE (TT, viscum or viscumTT) for 2 h. Cell viability was measured by LDH release assay. The results are expressed as a percentage of control ± SD (n = 3). There was no relevant LDH release (< 10% compared to control).

**Fig 2:**
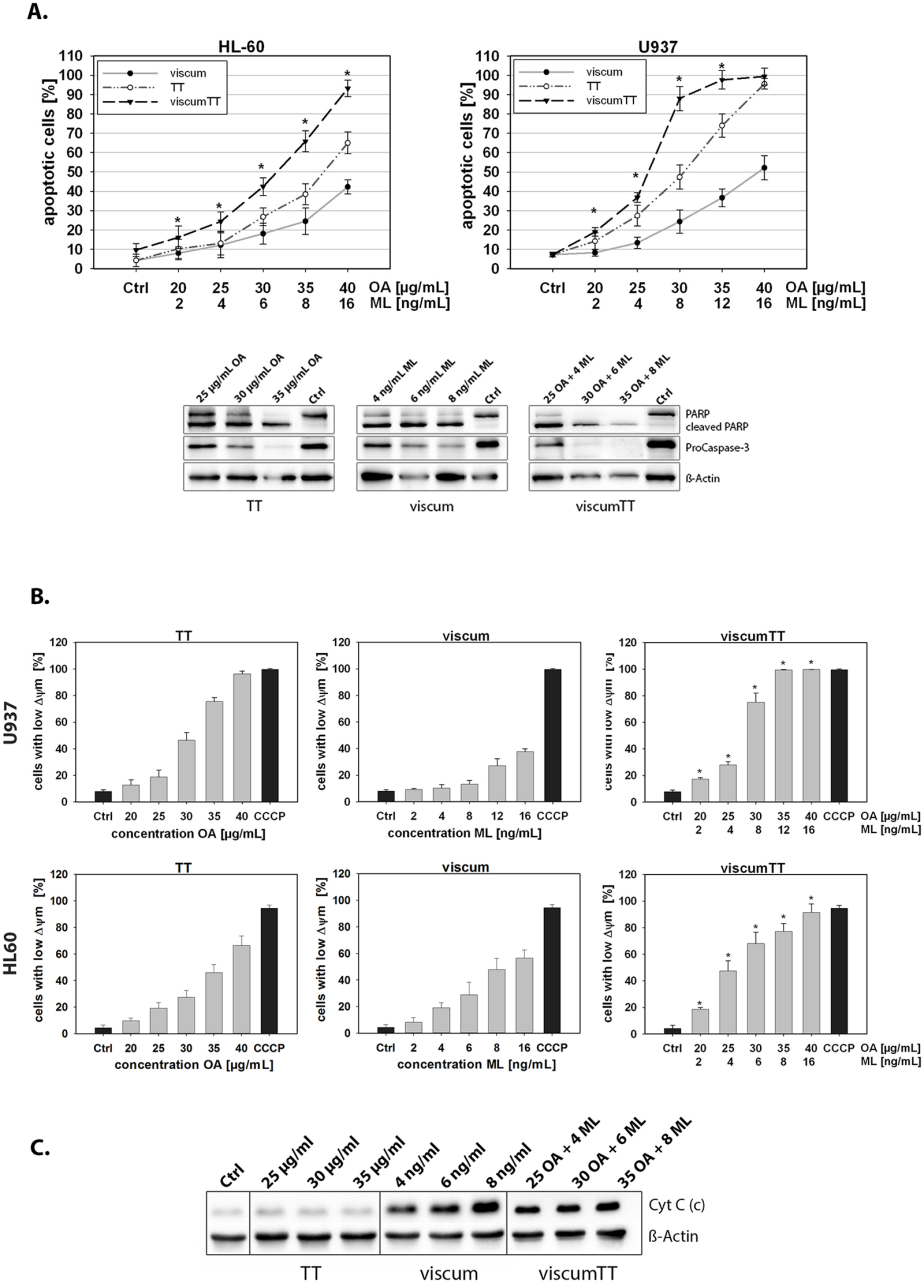
Triterpene acid- and lectin-containing extracts induce apoptosis in leukaemia cells. **A.** To measure the induction of apoptosis by VAE, U937 and HL-60 cells were treated with different concentrations of the extracts for 18 h. Apoptosis was determined with Annexin V-APC and propidium iodide by flow cytometry. Values are given as percentages of Annexin V-positive/PI-negative cells (± SD, n = 3). The results indicate a dose-dependent induction of apoptosis. Webb´s fractional product was used to calculate the additive, antagonistic or synergistic effect of the combination viscumTT. A single asterisk indicates Fp>1 and reveals a synergistic effect of viscumTT when compared to the single agents TT and viscum. Fp = fractional product. To confirm apoptosis induction after VAE treatment, whole cell lysates of VAE-treated HL-60 cells were separated by SDS-PAGE followed by Western blotting and decrease of procaspase-3 and cleavage of PARP was detected. Equal loading was verified using ß-actin as internal protein control. The blots are representative of three independent experiments. **B.** VAEs induce mitochondrial membrane permeability. U937 and HL-60 cells were incubated with TT, viscum or viscumTT for 18 h. After incubation, the mitochondrial permeability transition was measured by JC-1 and flow cytometric analysis on single cell level. Values of mitochondrial permeability transition are given in percentages of cells with low mitochondrial potential (ΔΨ_m_) ±SD, n = 3. A single asterisk indicates a synergistic effect of viscumTT compared to the product of the single agents (*Fp>1). The mitochondrial permeability transition did not change in the negative control. CCCP (carbonyl cyanide 3-chlorophenylhydrazone) was used as mitochondrial membrane potential disrupter (positive control). Both cell lines show a dose-dependent loss of mitochondrial membrane potential after VAE treatment indicating the involvement of intrinsic apoptosis induction. ViscumTT shows a synergistic effect. **C.** VAEs induce cytochrome c release from mitochondria to cytosol. Cytochrome c release was analyzed after 18 h of VAE incubation. Western blots display a dose-dependent release of cytochrome c from mitochondria to cytosol after treatment with TT, viscum or viscumTT. The adjusted density value of cytochrome c was quantified by Quantity One software (Density Ctrl = 1; n = 3). C = cytosol.

**Fig 3:**
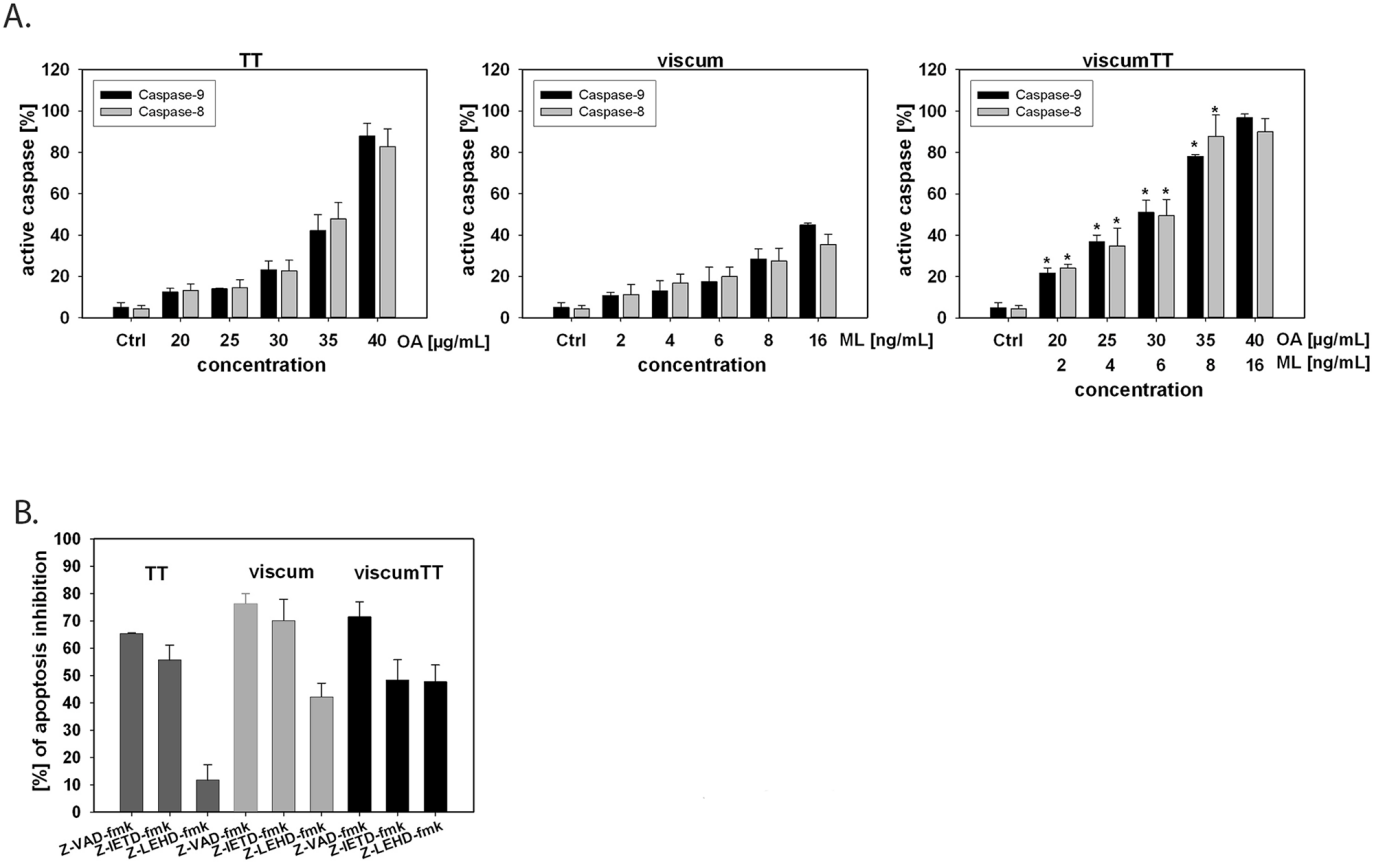
Caspase activity of HL-60 cells after VAE treatment. **A.** HL-60 cells were incubated for 18 h with VAE. Caspase-8 and-9 activity was measured by FITC-LEHD and FITC-IETD and flow cytometry, ±SD, n = 3. VAEs show a dose-dependent activation of caspase-8 and -9, viscumTT activates the caspases in a synergistic manner. * Fp>1, displays the synergistic effect. **B.** The cells were treated with TT, viscum or viscumTT for 18 h in the presence or absence of 100 μM z-VAD-fmk, z-IETD-fmk or z-LEHD-fmk. Effects of caspase inhibitors were analyzed by Annexin V/ propidium iodide staining and flow cytometry, ±SD, n = 3. Inhibition of caspase-8 reduces VAE-induced apoptosis by up to 55%, treatment with pan-caspase inhibitor shows an inhibition of apoptosis by up to 75%.

**Fig 4:**
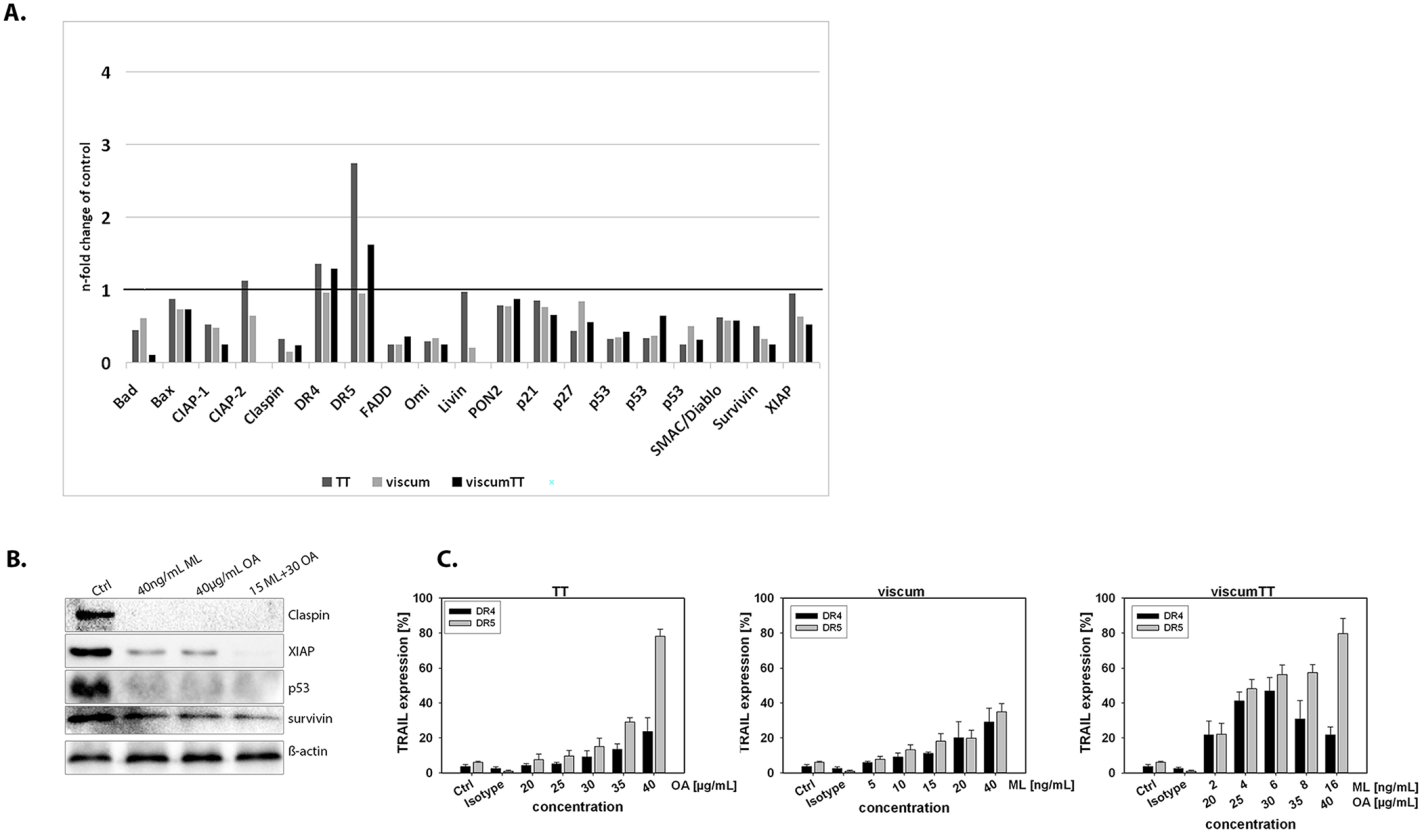
Expression of apoptosis associated proteins in HL-60 cells after VAE treatment. **A.** HL-60 cells were treated with depicted VAE concentrations (~IC50) for 18 h. Cell lysates were incubated with a human apoptosis array detecting the relative expression of apoptosis-related proteins per sample. Each experiment was carried out in duplicate (n = 2). VAE treatment changes the expression of apoptosis associated proteins resulting in an increase of TRAIL-R1 and a decrease in several proteins including XIAP, survivin, claspin and p53. **B.** The down-regulation of survivin, XIAP, p53 and claspin was confirmed by Western blot. **C.** Activity of TRAIL-receptor-1 and -2 was measured by FITC-labelled monoclonal antibody and flow cytometry after treatment of HL-60 cells with distinct VAE concentrations for 18 h.

**Fig 5:**
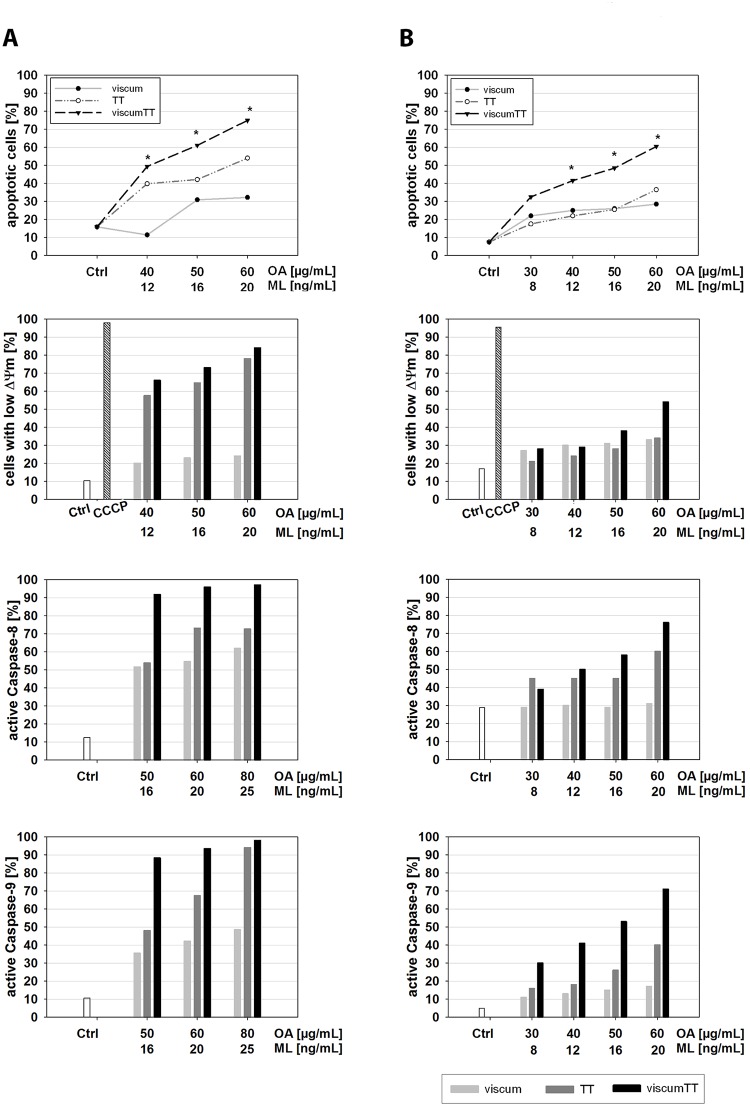
Mistletoe extracts induce apoptosis in acute myeloid leukaemia cells *ex vivo*. Mononuclear cells obtained from bone marrow aspirates of two patients with acute myeloid leukaemia (patient 1 (m; 17 years); patient 2 (m, 10 years)) were incubated with the depicted VAEs for 18 h. Induction of apoptosis (n = 2), mitochondrial transmembrane potential (n = 1) and activation of caspase-8 / -9 (n = 1) were measured by flow cytometry. VAEs induce apoptosis with involvement of mitochondria and activation of caspases in AML patients *ex vivo*, viscumTT shows synergistic apoptosis induction.

**Fig 6:**
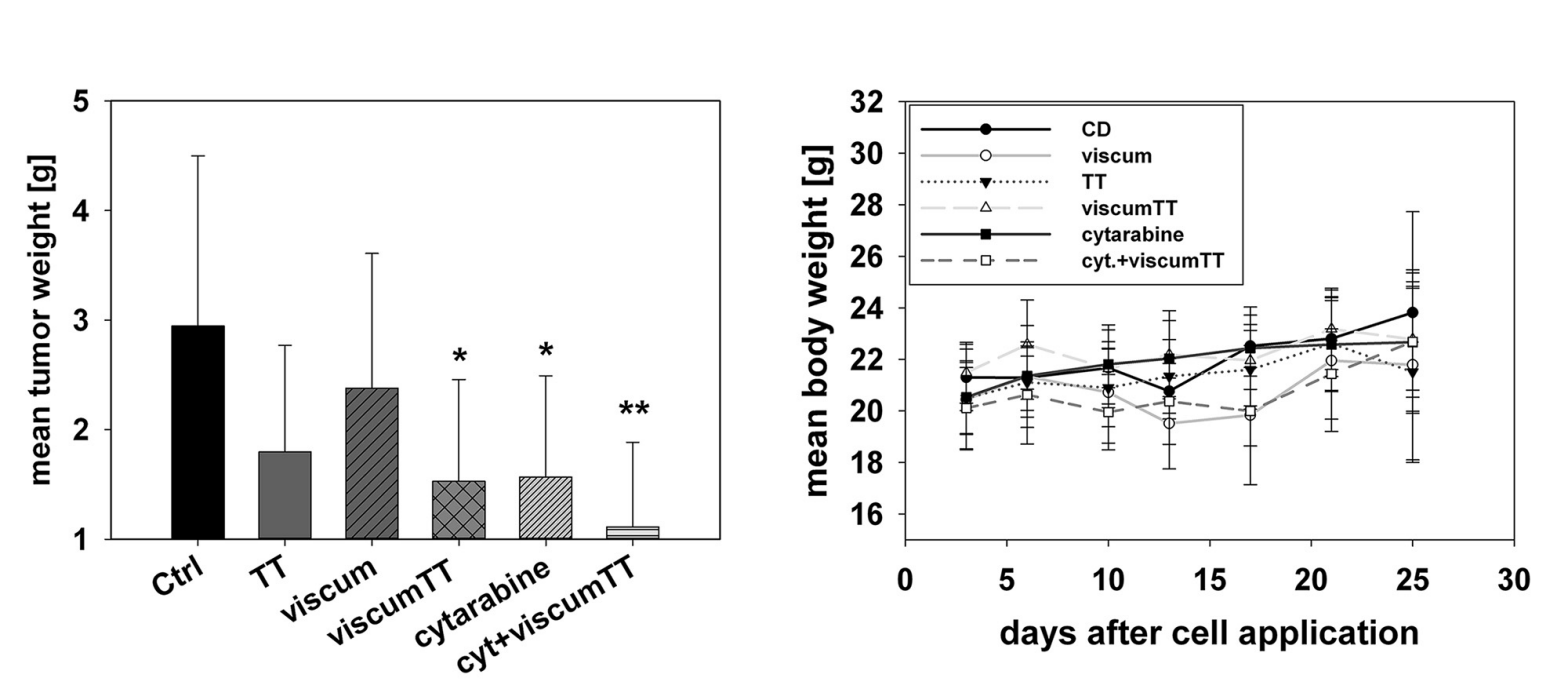
*In vivo* efficacy of *Viscum album* L. extracts. NOD/SCID/IL2rg mice carrying systemic human promyelotic leukaemia cells (HL-60) were treated with a dose-escalation regimen of 20/40/60/ mg/kg oleanolic acid (TT), 0.3/0.4/0.5 μg/kg lectin (viscum) or a combination thereof (viscumTT), three times per week for two weeks post-transplantation of cells. Each concentration were given twice (i.v.), starting on day three after tumor cell injection. One group was treated with cytarabine and one with cytarabine plus viscumTT. Control mice received equal amounts of cyclodextrins. The treatment with viscumTT had a significant therapeutic advantage compared to the single extracts TT and viscum.

The S1 Dataset represents the data underlying the results of Figs 1–6. These data are the basis of all presenting diagrams within this publication.

## Results

### Mistletoe lectins and oleanolic acid inhibit proliferation *in vitro*

To assess the anti-proliferative effect of an aqueous mistletoe extract viscum, an extract of cyclodextrin solubilised triterpene acids TT and the combination thereof viscumTT, U937 and HL-60 were incubated with increasing concentrations of VAE for 18 h. Cell proliferation rate was analyzed by CASY cell counter. In both cell lines TT and viscumTT inhibited proliferation markedly in a concentration-dependent manner, whereas viscum treatment did not lead to a significant inhibitory effect (Fig 1A).

To exclude an unwanted early cytotoxic effect via necrosis after VAE treatment, the release of LDH was measured after 2 h providing an accurate measurement of cell membrane integrity and cell viability. The percentage of total LDH released in the culture medium, as an index of necrotic cell damage induced by VAE, is shown in Fig 1B. Independent of doses, no marked release of LDH was measured with any of the three VAEs.

### Mistletoe lectins and oleanolic acid in combination induce apoptosis synergistically

To investigate whether apoptosis contributes to the anti-proliferative effect, the induction of apoptosis by viscum, TT or viscumTT in U937 and HL-60 cells was examined by annexin V-APC/PI and FACS analyses. Viscum, TT and viscumTT induced apoptosis in both cell lines dose-dependently (Fig 2A). Additionally, the incubation with the combination viscumTT induced apoptosis in a synergistic manner in both cell lines when compared to the single extracts viscum and TT. The synergism was calculated by Webb´s fractional product (*Fp>1).

To confirm the induction of apoptosis and to analyse the cell death in more detail, Western blot analyses were performed after VAE treatment in HL-60 cells. PARP, a known substrate of caspase-3-like protease in apoptosis, was cleaved into 89 kDa and 24 kDa fragments after VAE treatment, as expected (Fig 2A). For procaspase-3 a dose-dependent decrease was observed. These data indicate that VAE-induced apoptosis proceeds through caspase activation.

### Synergistic induction of mitochondrial depolarization and cytochrome c release

The reduction of mitochondrial transmembrane potential (MTP) accompanied by release of cytochrome c into cytosol is associated with the intrinsic apoptosis pathway. To measure the involvement of mitochondria in the induction of apoptosis, the MTP depolarisation in VAE-treated HL-60 and U937 cells was evaluated with the cationic dye JC-1. Treatment with TT resulted in a significant dose-dependent loss of mitochondrial transmembrane polarisation (Fig 2B), whereas viscum-treatment showed less effect on mitochondrial membrane permeability (MMP). Moreover, viscumTT caused significantly enhanced ΔΨ_m_ depolarisation and confirmed the synergistic effect observed in Annexin V assay.

In addition, cytochrome c was released from the mitochondria into the cytosol in response to VAE treatment (Fig 2C). Together, these results clearly indicate that both triterpene acids and lectins mediate induction of apoptosis via the intrinsic signalling pathway.

### *Viscum album* L. extracts affect caspase activity

For more detailed experiments, the activation of caspase-8 and -9 was assessed in VAE-treated HL-60 cells. Both caspases are upstream initiator protease caspases, which play key roles in the extrinsic (caspase-8) or the intrinsic (caspase-9) apoptotic pathway. The data in Fig 3A revealed a similar activation of both caspases by TT and viscum in a concentration-dependent manner. In addition, viscumTT-treated cells activated caspase-8 and -9 in a synergistic way as shown before (*Fp>1).

To differentiate the role of caspases in VAE-induced apoptosis, HL-60 cells were treated in the absence or presence of the caspase inhibitors z-VAD-fmk, z-IETD-fmk and z-LEHD-fmk. In TT-treated cells incubation with the caspase-9 inhibitor z-LEHD-fmk did not prevent apoptosis, whereas the caspase-8 inhibitor z-IETD-fmk inhibited the TT-induced apoptosis (55% inhibition) in nearly the same manner as the broad range inhibitor z-VAD-fmk (65% inhibition).

In viscum-treated cells, both z-IEDT-fmk and z-VAD-fmk were able to efficiently block the induction of apoptosis by 70–75% (Fig 3B). However, incubation with z-LEHD-fmk reduced the apoptosis rate by only 42%.

In viscumTT-treated cells, caspase-8 and -9 inhibitors blocked induction of apoptosis to a lesser extent (up to 48%) than z-VAD-fmk treatment (72% inhibition).

These results indicate that caspase-8 and -9 are involved and that both the extrinsic and intrinsic pathways play a role in VAE-induced apoptosis in acute myeloid leukaemia *in vitro*.

### VAE treatment changes expression of apoptosis associated proteins

To further elucidate the effect of VAE-induced apoptosis on apoptosis relevant proteins, a human apoptosis array was performed with HL-60 cells (VAE concentration ~IC50). We observed a decrease in inhibitor of apoptosis proteins (IAPs) cIAP-1, -2, livin, XIAP and survivin in TT, viscum and viscumTT—treated cells. Here, the most marked reduction of these proteins was observed after viscumTT-treatment (Fig 4A). Claspin, a protein which is required for efficient DNA replication during the S phase, also showed reduced expression in all VAE-treated cells. Furthermore, an increase in TRAIL-R1 (DR4) and -R2 (DR5) was detected, especially after viscumTT-treatment. Besides, a decrease in the mitochondrial pro-apoptotic proteins Bax, Bad, Omi/HtrA2 and Smac/Diablo and the apoptosis adaptor protein FADD was observed. Moreover, expression of the cell cycle inhibitors p21, p27 and p53 was reduced (Fig 4A). The down-regulation of survivin, XIAP, p53 and claspin was confirmed by Western blot for all three extracts (Fig 4B).

Taken together, these data indicate that inhibition of proliferation and VAE-induced apoptosis is mediated through down-regulation of apoptosis relevant proteins.

### VAEs induce death receptor 4 and 5 expression in HL-60 cells

DR4 and DR5 are apoptosis inducible receptors that contain a death domain which is involved in caspase-8 mediated apoptosis. Because of the caspase-8 involvement in apoptosis and the results of the human apoptosis array we examined the effect of VAE treatment on DR4 and DR5 surface expression with FACS analyses. Fig 4C shows a similar dose-dependent up-regulation for both receptors after TT treatment. For viscum we observed an increase in DR4 and DR5 expression of up to 20% with a marginally lower signal for DR5. For viscumTT a dose-dependent increase in DR4 expression was observed, whereas DR5 expression showed first up- and then down-regulation.

### Induction of apoptosis in patient samples after VAE treatment *ex vivo*

To investigate whether VAE treatment was able to induce apoptosis in primary tumour cells (*ex vivo)*, bone marrow aspirates of two patients with childhood AML were treated with increasing concentrations of viscum, TT and viscumTT. As shown in Fig 5, VAE treatment induced apoptosis accompanied by the loss of mitochondrial transmembrane potential in both *ex vivo* samples in a concentration-dependent manner. Moreover, we were able to confirm the synergistic effect of viscumTT *ex vivo* (*Fp>1). In addition, the measurement of active caspase-8 and -9 verified our *in vitro* data after VAE treatment.

### Therapeutic efficacy of viscumTT in AML/ NOD/SCID/IL2rg *in vivo*

To analyse whether VAE administration is able to inhibit growth of HL-60 *in vivo*, we designed a xenograft animal experiment. After administration, the tumour weight was compared with that in the control group. The results, shown in Fig 6, demonstrate that viscumTT-treatment reduced the tumour weight in a significant manner. Moreover, the tolerability of the administered VAE concentrations was very good. There was no evidence of toxicity measured by weight and autopsy including histology of liver, spleen and bone marrow. Just a minimal atrophy of the spleen was detected in one animal of the cytarabine group. Interestingly, viscumTT was able to reduce tumour weight in a similar manner to cytarabine. The combination of cytarabine and viscumTT showed the most significant effect with regard to tumour growth inhibition (p < 0.0053).

## Discussion

The data presented in our study indicate that all three VAEs exert anti-proliferative and apoptosis-inducing effects in a concentration-dependent manner *in vitro* and *ex vivo*. For viscumTT-treated cells we were able to demonstrate a synergistic effect on cell death induction. Moreover, in comparison to cytarabine-treated HL-60/NSG mice, viscumTT-treatment reduced the tumour weight in the same manner as cytarabine *in vivo*.

The *in vitro* results of this study show induction of apoptosis induced through the intrinsic and extrinsic signalling pathway. The involvement of the intrinsic signalling pathway for all tested extracts was evidenced by a significant loss of mitochondrial transmembrane potential, cytochrome c release from mitochondria and activation of caspase-9. The role of the intrinsic apoptosis pathway in mistletoe lectin I or triterpene acid-mediated apoptosis has also been shown by other authors [21, 25, 59].

However, it is known that triterpenes are able to induce apoptosis caspase-dependently or independently depending on the cell line [21, 37, 60, 61]. The results of the present study show activation of caspase-8, -9 and -3 in TT-treated cells, in line with our results in acute lymphoblastic leukaemia (ALL) cells [47]. In combination with the caspase inhibitor assay, where pre-treatment with z-VAD-fmk and z-IETD-fmk results in significant inhibition of apoptosis, the data indicate a caspase-dependent apoptosis for TT-treated HL-60 cells. Interestingly, beside the caspase-8 and broad-range z-VAD-fmk inhibitor, z-LEHD-fmk is also able to reduce the apoptosis rate in viscum and viscumTT- treated cells. Since the caspase-9 inhibitor only partly reduced induction of apoptosis by TT and viscum, we suggest that caspase-8 activation might be the major apoptotic signalling pathway. However, there is evidence that the extrinsic and intrinsic pathways are linked and that molecules in one pathway can influence the other. Activated caspase-8, for example, may directly cleave the pro-apoptotic Bcl-2 family member Bid for cross-talking with the intrinsic pathway [62]. This is in line with our results indicating an involvement of intrinsic and extrinsic apoptotic pathways.

In order to examine the mechanism of VAE-induced apoptosis, apoptosis relevant proteins were analysed by a human apoptosis array and Western blot analyses. Consistent with our flow cytometry results, the proteome profiler apoptosis array revealed up-regulation of DR4 and DR5 supporting involvement of the extrinsic apoptosis pathway. For OA and its derivatives such as CDOO, CDOO-Im and CDDO-Me it has been reported that they are able to induce TRAIL-dependent apoptosis [63–65]. To our knowledge, for viscum no impact on DR4 and DR5 surface receptor expression has been published to date [25].

Moreover, the proteome profiler apoptosis array displayed down-regulation of the pro-apoptotic Bcl-2 family proteins Bax and Bad and the apoptotic factors Htra2/ Omi and Smac/ Diablo. These proteins are involved in intrinsic apoptotic signalling and are released from mitochondria after apoptotic stimuli [66, 67]. The down-regulation may be the result of “usage” by binding IAP family members or anti-apoptotic proteins. Furthermore, our results indicate that several IAP family members (c-IAP1, XIAP, livin and survivin) are down-regulated by all three VAEs. IAPs are a family of proteins that are involved in cell death, immunity, inflammation, cell cycle and migration [68]. XIAP is a direct inhibitor of caspases, which binds caspase-9, -3 and -7. Cellular IAPs, e.g. survivin or c-IAP, by contrast, block the assembly of pro-apoptotic protein signalling complexes and mediate the expression of anti-apoptotic molecules [69]. Smac/ Diablo and Htra2/ Omi have been shown to block IAPs by binding [70].

For OA it was already shown in human non-small cell lung cancer cell lines and for the combination of OA and 5-FU in prostate cancer cells that OA-induced apoptosis was accompanied by a decreased protein level of survivin [71, 72]. For the triterpenoid CDDO-Me it was demonstrated that it is able to suppress NF-κB and NF-κB-dependent gene products that mediate cell survival, including survivin, Bcl-2 and cIAP2 in human leukaemia and ovarian cancer cells [73, 74]. Furthermore, treatment with CDDO-Me inhibited the Stat3 pathway with decreased levels of Bcl-xL, survivin and Mcl-1 proteins in multi-drug resistant osteosarcoma cell lines [75].

The activation of NF-κB can affect the TRAIL receptors DR4/DR5 and DR6, the death receptor Fas and the death-inducing ligands FasL, TRAIL, p53, Bax and the pro-apoptotic spliced form of Bcl-xL [76]. Further studies are needed to evaluate whether NF-κB is one of the major signalling pathways for TT or viscumTT-induced apoptosis in acute leukaemia cells. However, to our knowledge this is the first time that the status of survivin has been analysed in mistletoe lectin I treated cells. The role of these proteins will need to be further characterized in VAE-treated cells.

Furthermore, we detected a decrease in claspin in all three extracts. Claspin is an essential upstream regulator of checkpoint kinase 1 and initiates a checkpoint arrest of the cell cycle in response to replicative stress or DNA damage. It has been shown that claspin positively affects the survival of cancer cells [77]. The decrease in this protein may play a role in the VAE-induced inhibition of proliferation.

Additionally, p53, p21 and p27 were reduced in TT, viscum and viscumTT-treated HL-60 cells. HL-60 cells have a mutated p53 which leads to loss of function, yet truncated forms of p53 can still exist in the cells. The tumour suppressor protein p53 responds to diverse cellular stresses and regulates the expression of target genes, thereby inducing cell cycle arrest, apoptosis, senescence, DNA repair, or changes in metabolism. P21 and p27 are cyclin-dependent kinase inhibitors both of which function as regulators of cell cycle progression in a p53-dependent or independent way [78]; [79, 80]. Short chain fatty acids (SCFA) have been shown to induce p21 and p27-mediated cell growth arrest with down-regulation of p53 and induction of apoptosis by activation of stress mediated JNK signalling in colon cells [81]. Interestingly, Büssing *et al*. reported a reduction in p53 expression after ML-III treatment [82]. The activation of the JNK pathway by VAE and the role of p53 have not yet been investigated.

The potent *in vitro* activities of viscumTT prompted analysis of the anti-cancer effect *in vivo*. For mistletoe lectin I, oleanolic acid and its derivatives an anti-metastatic effect has been already described *in vivo* [23, 26, 71, 83–85]. Furthermore, an anti-cancer effect of the combination viscumTT has been reported by our group in an ALL model and by Strueh *et al*. in a melanoma mouse model [47, 48]. In the present study viscumTT-treatment reduced the tumour weight in a significant manner *in vivo*. More interestingly, the viscumTT-treatment reduced the tumour weight in a similar manner to cytarabine-treatment. These findings confirm data published earlier and, moreover, show the effectiveness of viscumTT compared to cytarabine. Combining this with the *in vitro* results, we suggest that the overall therapeutic effect and induction of apoptosis by viscumTT derived from several compounds acting tog8ether synergistically. Treatment with TT or viscum alone reduced tumour weight in HL-60/NSG, though we could not show significance in tumour reduction. It is noteworthy that our results suggest that a substance combination occurring in the mistletoe plant represents a kind of phytotherapeutic polychemotherapy.

Furthermore, the additional use of viscumTT strongly potentiates the anti-tumour effect of cytarabine. A similar result has also been reported for the Japanese apricot extract (containing triterpenes), which amplified the antineoplastic activity of 5-FU [86]. To our knowledge, no *in vivo* data about the efficacy of an aqueous mistletoe extract with a similar therapeutic effect to a classical cytostatic have been published to date.

In summary, we were able to show that the combinatory extract viscumTT combining aqueous mistletoe compounds and triterpene acids induces apoptosis in HL-60 cells via the intrinsic and extrinsic signalling pathways.

The *in vivo* study displayed a strong therapeutic effect for viscumTT comparable to that of cytarabine. The potentiating effect of viscumTT and cytarabine provides strong evidence for combination with classical chemotherapy agents to achieve a high synergism of effectiveness in cancer treatment.

## Supporting Information

S1 Dataset Minimal data set underlying the findings of Figs 1–6. (XLS)
